# Magically deceptive biological motion—the French Drop Sleight

**DOI:** 10.3389/fpsyg.2015.00371

**Published:** 2015-04-09

**Authors:** Flip Phillips, Michael B. Natter, Eric J. L. Egan

**Affiliations:** ^1^Vision and Haptics Laboratory, Neuroscience and Psychology, Skidmore CollegeSaratoga Springs, NY, USA; ^2^The Vision Laboratory, Department of Psychology, The Ohio State UniversityColumbus, OH, USA

**Keywords:** magic, perception, biological motion, deception, deception detection

## Abstract

Intentional deception, as is common in the performance of magic tricks, can provide valuable insight into the mechanisms of perception and action. Much of the recent investigations into this form of deception revolve around the attention of the observer. Here, we present experiments designed to investigate the contributions of the performer to the act of deception. An experienced magician and a naïve novice performed a classic sleight known as the French Drop. Video recordings of the performance were used to measure the quality of the deception—e.g., if a non-magician observer could discriminate instances where the sleight was performed (a deceptive performance) from those where it was not (a veridical performace). During the performance we recorded the trajectory of the hands and measured muscle activity via EMG to help understand the biomechanical mechanisms of this deception. We show that expertise plays a major role in the quality of the deception and that there are significant variations in the motion and muscular behaviors between successful and unsuccessful performances. Smooth, minimal movements with an exaggerated faux-transfer of muscular tension were characteristic of better deception. This finding is consistent with anecdotal reports and the magic performance literature.

## 1. Introduction

Science and magic live on opposite ends of the empirical spectrum. The scientific community relies on a controlled, methodological approach as its guiding principle whereas the magician's motivation rests on the art of deception, frequently by denying legitimate observation. Yet it comes as no surprise that magic provides a fertile ground for the scientific study of perceptual and cognitive processes. Magic plays off of the intuitive rational sense of human cognition. Sleights of hand require skill, dexterity, and coordination, and are thus rooted in psychological phenomena that stem from biophysical foundations. This makes it possible to study specific illusionary actions in a psychological and/or neuropsychological scope to better understand deceptive biological motion and its mis-perception (Binet, 1894; Jastrow, 1896; Hyman, 1989; Kuhn et al., 2008; Macknik et al., 2008; Lamont and Henderson, 2009).

Magic relies on a broad set of mechanisms and processes to carry out its illusory effects. These include mechanical or physical manipulation (e.g., the deformed position of the assistant, facilitated by the “special” table, in Selbit's “Sawing Through A Woman”) as well as psychological and cognitive manipulation or exploitation (e.g., the assumption of good continuation of the aforementioned assistant). Successful illusions will involve some combination of these. Investigations of these mechanisms use an equally broad range of techniques, focusing on the social and attentional cues that accompany such illusions (e.g., Kuhn and Land, 2006), the perceptual mechanisms involved in deception (e.g., Barnhart, 2010), perceptual-motor mechanisms (e.g., Cavina-Pratesi et al., 2011) and the underlying neuropsychological mechanisms (see Macknik et al., 2008, for an extensive review).

The universe of events and techniques that constitute the realm of “magic” is extensive. The domain of sleight of hand magic provides a constrained and well defined behavioral and experimental environment in which to explore these processes and mechanisms. For example, Cui et al. (2011) have used this paradigm to investigate the attentional behavior of the audience, showing that social cues may not be necessary to effectively convey deception. Of course, there are two parties involved in these magical transactions—the deceiver and the deceived. Jastrow (1896) performed a series of tests on sleight of hand magicians to determine if they had perceptual and mechanical skills “above and beyond” that of the lay public. Indeed, for the limited sample available several differences appeared, some positive (auditory sensitivity, simple reaction time) but others were the same or negative (complex reaction time, acuity, tactual perception). More recently Otero-Millan et al. (2011) investigated the deceptive qualities of motions, focusing on the performers' contributions to the deception. In this spirit, our interest lies in the entire interaction of performer and audience. What aspects of deceptive biological motion are controlled by the performer and what parts are the audience's share?

So-called “misdirection” is the fundamental platform on which sleight of hand magic rests. The magic literature frames misdirection as a method of controlling the observer's attention (Nelms, 1969/2000; Lamont and Wiseman, 2005) and suggests several techniques for achieving it. As suggested above, this attentional control can arise from a variety of sources, ranging from overt social cues (“Hey! Look over there!”) to subtle, practiced, and precise perceptual-motor manipulations. Thus, magic can help us disentwine how the *performance* of the action contributes to the *perception* of that action. To properly do so, one must isolate and examine the physical mechanism of the deception to understand and identify the psychophysical characteristics of deceptive biological movements. Johansson (1973) presented a framework for understanding the perception of biological motion that has resulted in a number of studies by Troje and others Troje (2002); Troje et al. (2005) on the use of biological motion information for identification of identity and intent. The field of sports-science has embraced this technique, typically to study anticipation in competitive scenarios (Müller et al., 2006; Abernethy, 2008; Huys et al., 2008; Possidente et al., 2011; Diaz et al., 2012) and, by extension, the nature of deceptive motion (Farrow and Abernethy, 2003; Jackson et al., 2006).

Along with intentional misdirection, it is instructive to consider the effects of dynamic occlusion and predicted outcome location. Wexler and Klam (2001) highlight the gestalt principle of good continuation (also see (Barnhart, 2010) and its prevalence when viewing illusionary movement. Perceptual behavior consistent with good continuation is present from infancy (Quinn and Bhatt, 2005), suggesting that this assumption may be responsible for some of the illusory phenomena found in prestidigitation. Similarly, Soechting et al. (2001) address deceptive movement and anticipated location. Given the findings that a moving background affects the perceived direction of a target in motion (e.g., the Duncker Illusion), participants were asked to follow a target moving in a straight line, which became occluded by a band of randomly moving dots, and point to the predicted outcome of the line. The expected pointing errors correlated with the Duncker illusion. The participant's eye movements were concentrated in the lower border of the occluded area once the target vanished and attempted to maintain fixation in this zone. Due to the random horizontal movement of the occlusion dots, fixation from the desired lower border was altered which correlated to pointing errors. This amodal completion-like effect is also present temporally in magic performances that involve deceptive transfer of items from hand to hand (Beth and Ekroll, 2014).

Finally, it is informative to examine the broader intention of biological movement (Michotte, 1963; Király et al., 2003). One such study examined the recognition onset of sign language across deaf signers, hearing signers, and non-signers (Arendsen et al., 2007). The results show that the intention of sign language gestures can frequently be derived solely from the initial hand motion. Given this, we predict that the initial phases of a deceptive motion may also incorporate information necessary for identifying deceptive intent.

What are the quantifiable differences between veridical and deceptive motion in sleight of hand magic and can we tease out the deceptive characteristics?

## 2. The French Drop Sleight

**Figure 1:**
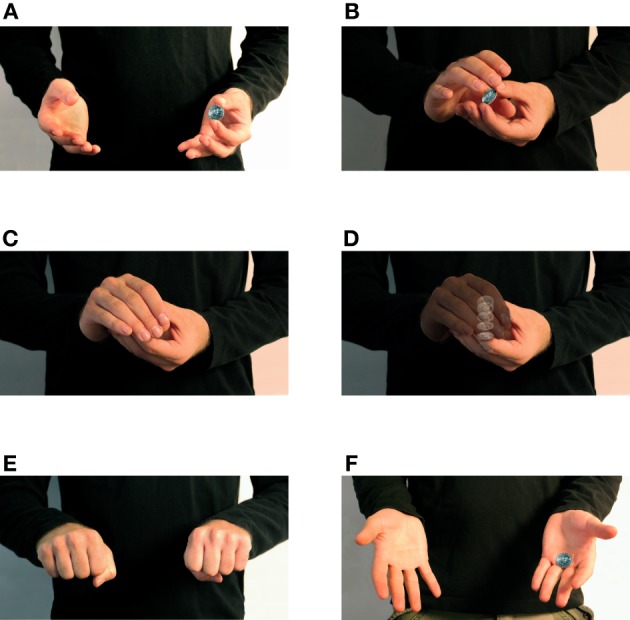
**Performance of the French Drop Sleight. (A)** Starting position of the French Drop sleight. The object to be “vanished” is held in one hand, with the both hands held in an open fashion to demonstrate to the viewer that there is nothing concealed. **(B)** The empty hand is brought to the gripping hand, typically with the thumb and index finger positioned as if to grab the object. **(C)** In the typical performance of the FDS the object is concealed, as if it were grabbed by the fingers and thumb of the approaching hand. **(D)** In reality, the coin is dropped into the palm of the gripping hand, simultaneously with a faux grasping motion of the approaching hand. In our experiments, depending on the stimulus condition, the coin was either *actually* grabbed (the “veridical” condition) or was dropped (the “deceptive” condition). **(E)** The hands are separated and the observer is asked to chose which hand is presently holding the coin. **(F)** The actual location of the coin is revealed.

A commonly used magic sleight of hand illusion known as the French Drop Sleight (FDS) is used for the current study. Successful performance of the FDS results in the illusion of a small object vanishing. The illusion is created by starting with a small object (typically a coin) in one hand, while the opposite hand approaches and connects, appearing as if the object is being grasped, while actually maintaining the coin in the original hand, demonstrated in Figure 1.

The deception is achieved by covertly dropping the object from the thumb and forefinger of the initial hand, into the palm as the empty hand masks the drop by appearing to grab the object.

In reality there are two possible outcomes of this action: a veridical situation, where the object is *actually* transferred from one hand to the other, and a deceptive one where the object remains in the original hand.

When performed convincingly, this illusion is thought to be effective for two primary reasons: (1) social cues and automatic preconception, (2) instinctual gestalt principles applied to the motion Nelms, (1969/2000). In the case of (1), an onlooker, unsuspecting of the FDS about to be performed, viscerally assumes the coin is going to be transferred between hands due to it being the most overt and cognitively logical outcome given the visual information presented. Effect (2) suggest that, when presented with a motion that entails partial obstruction, as is the case with FDS, the brain instinctively applies the gestalt principle of good continuation to aid in filling in the gaps omitted from the visual field (Quinn and Bhatt, 2005; Barnhart, 2010; Beth and Ekroll, 2014). Thus, a skilled magician takes advantage of this automatic process by performing the FDS in one fluid motion instead of its constituent phases.

In addition to the above quantifiable mechanisms, a third mechanism is postulated, that of the transfer of muscular tension between the two hands (Teller, Personal Communication). The tension of one's hand when holding a coin is markedly rigid when compared to the free hand and this can be exaggerated for effect. This is thought to be exploited by the magician as he appears to take the coin. His hands transfer the tension (but not the coin) across hands, further cementing the illusion of the coin being exchanged.

## 3. Experiment 1

To effectively use the FDS as a model of biological deceptive motion it is first necessary to assess the salience of the sleight itself. Here we use a signal detection based technique to quantify its detectability.

To investigate skill-related variations our experiments use two magicians, a complete novice as well as an experienced performer. By noting variation between skill levels we hypothesize that salient elements of the deception are revealed by comparison.

### 3.1. Method

An expert and novice magician were filmed performing the FDS with two outcomes. First, a deceptive condition where the coin was not transferred between hands, and second, an equivalent veridical condition where the coin was transferred. Subjects were instructed to watch each film clip and respond by indicating which hand they thought the coin was in at the finish.

#### 3.1.1. Subjects

A total of 13 subjects participated in Experiment 1. All were Skidmore College students and received credit toward the research requirement of their Introductory Psychology course.

#### 3.1.2. Stimuli

The stimulus material consisted of 68 movie clips. These movie clips were filmed using two different skill-levels of magicians—a novice and an expert.

The expert has been performing the FDS for 10+ years while the novice had not performed the FDS before this experiment. There are numerous variations and styles of the FDS, therefore the expert magician trained the novice the mechanics of the maneuver and provided critical observation during a 1-week learning period. This ensured that the motions of the two magicians were similar at least at a coarse level. Both performers had the same dominant hand (right).

There are significant social cues and misdirection that can be employed to enhance the performance of a successfully deceptive FDS. For example, imploring the observer to keep a close eye on one hand or the other serves to direct or misdirect attention. Further deception can take place via head and eye movement of the magician, again directing the attention away from where the “business” of the trick is taking place. Since we are interested solely in the biological motion aspects of the FDS we have removed these potentials for social cuing in this and the following experiments.

Each magician wore a long sleeved black shirt and performed in front of a black backdrop. The image frame was cropped such that only the chest, arms, forearms, and hands were visible (See Figure 1 for an example of the framing). During filming, the magicians performed 20 repetitions of the FDS as well as a veridical variation of the motion where the coin is actually exchanged into the implied hand. Of the 20 repetitions, the amateur dropped or mishandled the coin on three takes, resulting in 17 usable performances. We took the first 17 usable takes from each performer in each condition for a total of 68 clips.

The clips were then edited using iMovie (Apple Inc.) to exclude any extraneous motion at the beginning and end and a two second black buffer was added pre- and post-clip as well as a two second “respond now” screen to allow for the subjects' response. Each clip averaged 8 s, including the buffer and response cue, and had no sound track. The final stimuli were rendered as 640 × 480 movies at 29.97 fps, compressed using the Quicktime (Apple Inc.) “Video” compression codec in high quality.

Figure 1 illustrates the extent of the motion shown in each trial.

The 68 clips were presented twice, in two blocks with a brief break between. In the first block subjects were shown the result of the trial after responding. We refer to this phase as the “reveal” as demonstrated in Figure 1F. This provided the subject with feedback as to the accuracy of their response so as to establish best-performance as well as to facilitate learning any “tells” or consciously detected cues that would facilitate the detection of the sleight. In the second block subjects were shown the same set of 68 clips but not shown the reveal. In both blocks the conditions were fully randomized across performer and condition.

Examples of the performance clips can be seen at http://vimeo.com/user20016520/fds.

#### 3.1.3. Procedure

The subject was seated approximately 57 cm from a 58 cm (23”) iMac (Apple Inc.). No chin-rest was used, thus observers had free motion of their heads. The video clips of the performance took up the entire screen. They were presented with a written explanation of the experiment as well as verbal reiteration from the experimenter. Subjects were instructed to view each clip and respond by indicating which hand they believed the coin was in. Responses were recorded by the participant on a printed response sheet. They were shown the first block of 68 trials (featuring the “reveal” feedback), followed by a short break, then shown the second block, without feedback.

## 3.2. Results and discussion

A comparison between the feedback and no-feedback conditions, using Wilcoxon's signed-ranks, shows no difference in detection across the within-performer conditions, *W* = 28, *p* = 0.41 for the novice and *W* = 27, *p* = 0.62 for the expert. This further demonstrates that no significant learning takes place via the feedback of the “reveal.” This suggests that, at least for these presentation conditions, whatever information used for making decisions about the presence or absence of the coin was readily available.

Observers detected the correct ending hand for the novice's performance an average of 74.2% of the time *S.E*. = 3.6% with *d*′ = 1.18, 95% CI [0.91, 1.45], a moderately effective detection performance. On the other hand, detection for the expert performance was only slightly above chance at 55.9%, *S.E*. = 7.7% with *d*′ = 0.32, 95% CI [0.17, 0.51]. Thus, as would be intuitively expected, subjects are much better at determining the outcome when the FDS is performed by the novice, as opposed to the expert.

The detection criterion is negative in both cases, *c* = − 0.17, 95% CI [−0.22, −0.11] for the novice performer and *c* = −0.43, 95% CI [−0.57, −0.29] for the expert. This shows a response bias *toward assuming deception* in veridical presentation conditions. More specifically, judging that the coin is *not* taken when in fact it is. Thus, subjects assumed deception across both performers. While this is not terribly surprising—that observers watching a potentially deceptive performance are predisposed to assume deception—the bias is strongest in the expert presenter condition. Since we only used two performers it is possible that the observers internalized the stereotypical motion or some other cue, such as characteristics of the hands, during the initial block with the reveal. Subsequently, these cues may have indicated that an effective performance was afoot and the observers assumed deception.

## 4. Experiment 2

The results of Experiment 1 establish the strength of the illusion as well as the effect of expertise on its performance. These results are not particularly surprising—they confirm our intuition and phenomenological experience of the deception and the effect of the caliber of the performance. This established, our remaining experiments probe the nature of the motion and the potential cues that serve to cause the deception.

We first investigate the individual phases of the motion so as to establish at what point the deception tends to takes place. Arendsen et al. (2007) have used sign language gestures, broken into naturally defined phases. The salience of the global sign is then evaluated during their isolated (e.g., partial) presentation. The current experiment adapts this technique. We divide the full-motion stimuli of Experiment 1 into three phases defined as: approach, capture, and retreat. As in our previous experiment, subjects watch each clip and respond by indicating which hand they expected the coin to end in.

### 4.1. Method

The method used in Experiment 2 is identical to that of Experiment 1—Clips of the FDS performance were shown and subjects were told to predict the hand the coin would result in. However, different stimuli were used- partial clips of the motion representing one of three phases of the overall FDS instead of the original clips of the whole motion.

#### 4.1.1. Subjects

A total of 21 subjects participated in Experiment 2. All were Skidmore College students and received credit toward their Introductory Psychology course. One subject was excluded due to extensive errors in recording responses, leaving 20 subjects.

#### 4.1.2. Stimuli

**Figure 2:**
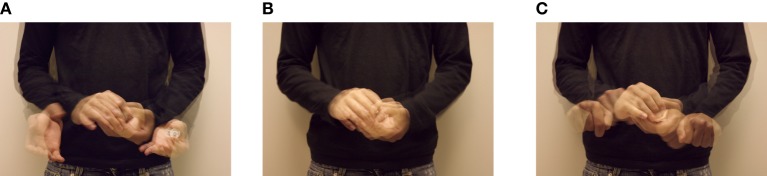
**The three phases of the FDS. (A)** The approach phase consists of the motion from the initial presentation to the preparation to grasp (or perform the grasp deception) the object, e.g., the progression from Figures 1A,B. **(B)** The capture phase consists of the portion of the motion from the preparation to grasp to the occlusion of the object, e.g., the progression from Figures 1B,C. **(C)** The retreat phase consists of the motion from the object's occlusion to the finishing hand positions, e.g., the progression from Figures 1C–E.

Experiment 2 uses the performance stimuli from Experiment 1 without the feedback (e.g., “reveal”) after the postcapture retreat phase. As with Experiment 1, performances from both novice and expert performers are used. These 68 clips are split into three phases of motion—the approach, the capture, and the retreat, illustrated in Figure 2. This resulted in a set of 204 movie clips. The three phases characterize the motion—inflection—motion sequence.

Across performers, conditions and performances the motion took *x* = 3.2, *s* = 0.2 s from the onset of the approach to the end of the retreat. The capture phase (from the initial obscuring of the coin until the separation of the hands) took an average of 0.9 s across performers and conditions.

To create the individual clips, the onset and termination of the motion were marked in the time-coded video, then transition time points were established by centering a 0.9 s window over the capture phase. The average location of these events as observed by the three authors and an additional lab member were used to define the three phases.

Figure 2A illustrates the approach phase, consisting of the motion of the hands from the start position to the position immediately before the two hands begin to overlap. The discrete positions are shown in Figures 2A,B respectively. Figure 2B shows the capture phase, consisting of the portion of the motion where the two hands overlap, either grabbing the coin or performing the deception. The discrete positions of the capture phase are shown in Figures 1B,C. Finally, Figure 2C shows the retreat phase, consisting of the motion from the end of the grabbing motion to the finish position. These positions are shown in Figures 1C–E.

#### 4.1.3. Procedure

To familiarize the subjects with the FDS they were first shown a demonstration set of 12 full-length performances. These performances included the veridical and deceptive conditions, performed by the novice and expert magician including the reveal. They were then instructed that they would see pieces of the motion and were told to predict which hand they expected the coin to end up in at the end of the motion. Since Experiment 1 showed no effect of feedback all trials were run without revealing the actual result.

The 204 trials were broken into two blocks of 102 clips with a short break provided between blocks. Responses were recorded by the subject manually as in Experiment 1.

### 4.2. Results and discussion

**Figure 3:**
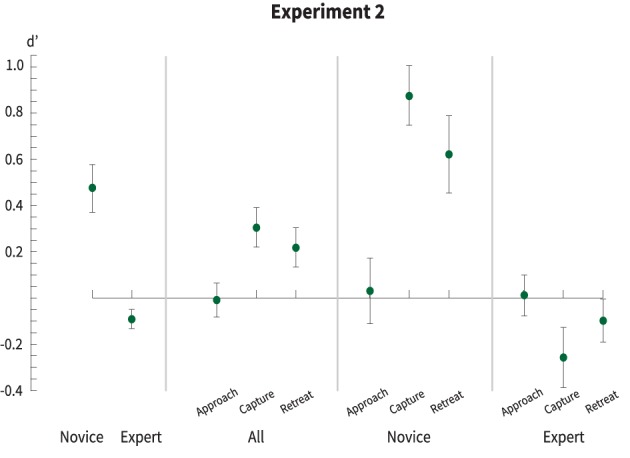
***d*′s by presenter and phase of motion, lower values indicate a more successful deception**. Error bars show the 95% CI. As with Experiment 1 there is a clear difference between the efficacy of the novice and expert performer, overall. When the motion is broken down into its constituent parts we see a clearer delineation. Overall, detectability was roughly at chance for the approach-phase but improved during the capture- and retreat-phases. The differences between the expert and novice performer are extreme—the expert is able to deceive with nearly no detection, while the novice reveals the location, most saliently in the capture-phase, but also in the retreat-phase as well.

The resulting *d*′ for Experiment 2 are shown in Figure 3. Overall, and as with Experiment 1 there is a clear difference between the novice and expert magician.

Overall, as with Experiment 1, the experience of the performer had a significant effect on detection (*d*′_*novice*_ = 0.5, *d*′_*expert*_ = −0.1) but, the overall detectability decreases since subjects are only shown “snippets” of the extended trick. A repeated measures ANOVA shows a significant effect of expertise [*F*_(1, 114)_ = 7.49, *p* < 0.01, η^2^ = 0.50] and an interaction between expertise and motion-phase [*F*_(2, 114)_ = 3.1, *p* < 0.05, η^2^ = 0.22].

There is no effect for either the novice or expert magician during the approach phase of the motion, (*d*′ = 0) for both performers. The capture phase, however, yielded a significant effect with the expert magician eliciting more false alarms among participants (*d*′ = −0.2) and the novice inducing a higher percentage of hit responses (*d*′ = 0.88), reinforcing the effect for skill level as well as highlighting the phase which contains the most variance across magicians. The novice, to a lesser degree, also elicits a higher sensitivity among participants during the retreat phase, while the expert remained at chance levels during this phase (*d*′ = 0.07). Therefore, it is likely that the expert performed the trick with the same motion, regardless of condition, where the novice “showed his hand” not only during the actual “move” (e.g., coin exchange) but afterward as well.

What is it about the post-move motion that gives the trick away?

## 5. Gross hand motion and grasp force

Experiments 1 and 2 demonstrate an effect for the performers' skill level and identify the segment of the motion that accounts for the largest difference in deceptive ability between the novice and expert performer.

We would next like to explore the characteristics of the motion that serve to induce this deception. Cavina-Pratesi et al. (2011) have shown that, when the object to be grasped is present (e.g., not absent with the grasp pantomimed), the grasp motions during a deceptive performance closely match those of veridical performance of the task. Our previously described experiments use a single novice and a single expert magician, making a statistically sensitive assessment of generic differences between novices and experts impossible. Still, it is informative to examine characteristics of the performers' kinematic and muscular differences in the hope that they may elucidate some aspect of the performances that differentiate the skill levels.

### 5.1. Gross hand motion

We first examine the global trajectory of the hands during the performance of the FDS. We hypothesize that the motion of the expert will be more consistent, as suggested by (Cavina-Pratesi et al., 2011), regardless of deceptive or veridical presentation. The novice should exhibit more variability and, potentially, inconsistency between the two presentation conditions.

#### 5.1.1. Apparatus and material

To gather position and pose during the FDS motion, a Polhemus 3Space Isotrak II (Pohlhemus, Inc.) motion tracking system was utilized. This is a 6-axis system, capable of providing position {*x*, *y*, *z*} and pose {*pitch*, *roll*, *yaw*} information at a temporal resolution of 60 Hz, an angular resolution of 0.1°, and a spatial resolution of 0.5 cm. Position and pose was acquired from the Isotrak via a USB-serial port converter, using an Apple MacBook Pro running Mac OS X.

The performing magician was outfitted with the Isotrak transmitter unit on the topside of the “working” (the gripping, right) hand. The corresponding cable was secured to the forearm using Velcro bands to prevent interference with the motion. The same large white coin was utilized during the performance of the FDS as in the previous experiments.

#### 5.1.2. Procedure

Each magician performed twenty deceptive and twenty veridical trials in a random interleaving. By randomly specifying the trials we hoped to avoid a patterned, stereotypical motion as a result of repetitively performing the same task.

### 5.2. Results and discussion

**Figure 4:**
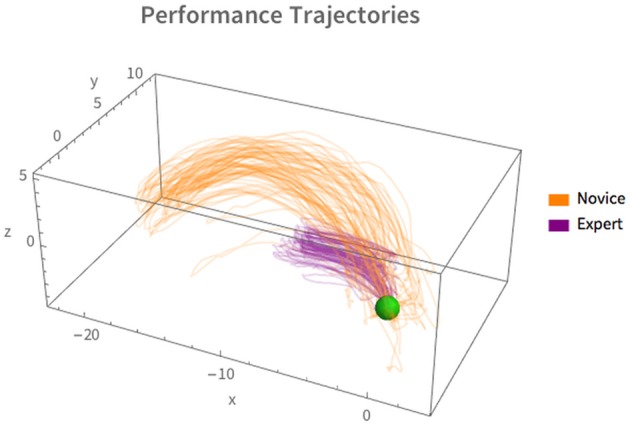
**Trajectories of the “working” hand for novice (orange) and expert (purple) performers, collapsed across performance type (e.g., deceptive and veridical) and viewed from in “front” of the performer**. All units are cm. The green sphere represents the position of the hand at the start. The spread of trajectories for the expert show a more economical set of trajectories, while the novice move a greater distance and has a characteristic arc not present in the expert's trajectory.

Figure 4 shows the overall trajectories of the working (e.g., right) hand for both performers, novice in orange and expert in purple. The green ball represents the beginning of the move. The difference in trajectories is qualitatively clear—the expert uses a more compact, less variable, linear motion whereas the novice has a broader, more variable motion that consists of a considerable arc. Indeed, sometimes exaggerated features of a performance add more “presence” and, often times, more “reality” to a performance (For an example from the world of animation, see Thomas and Johnston, 1981, where they discuss the effects of exaggeration on the perception of realistic movement). However, as shown in Experiments 1 and 2, the performance of the novice was not convincing, and therefore the exaggeration likely proved more of a distraction than an enhancement.

To successfully carry out the FDS it is important that the motor performance not belie the true location of the coin. Therefore, there should be no perceptible difference between the deceptive and veridical conditions. If, on the other hand, there is a perceptible difference between trajectories the subjects could use those differences inform their judgments.

To investigate this, we computed the variance in the working-hand trajectories using a Principal Component Analysis (PCA) based technique, similar to the methods of Todorov and Jordan (2002) and Diaz et al. (2012). Briefly, the trajectory is normalized in time such that each trial takes place on the interval *t_norm_* = [0, 1]. This normalization means that the approach-phase begins at *t_norm_* = 0, the capture-phase occurs around *t_norm_* = 0.5, and the retreat-phase finishes by *t_norm_* = 1.0. The normalized trials are resampled using linear interpolation and the resulting hand-position *x*, *y*, *z* coïdinates subjected to PCA. The variability of the derived components is then computed between performance conditions over the time course of the motion.

**Figure 5:**
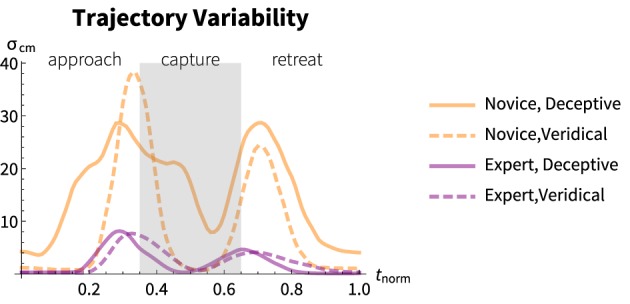
**Total variability across the timecourse of the normalized motion**. The first principal component is shown for each magician and each performance condition, orange and purple representing the novice and expert as in Figure 4. Solid lines represent the deceptive presentation and broken lines represent the veridical condition. The “capture” phase is denoted by the gray background, the approach to the left and the retreat to the right. On average the novice shows much more variability between trials than the expert. The expert shows less variability between trials between conditions than the novice.

For both expert and novice, veridical and deceptive conditions, >99% of the variance was accounted for by the first principal component. A summary of the variability accounted for by this component over the course of the motion is shown in Figure 5.

Across the timecourse of the motion the novice showed significantly greater variability, on average, than the expert (*U* = 33,925, *p* < 0.001, *r* = 0.81) with a median variance of *Mdn_novice_* = 9.94 cm and *Mdn_expert_* = 1.48 cm. For the novice performer there is a significant difference in variability between presentation conditions (*U* = 7668, *p* < 0.0001, *r* = 0.61) whereas for the expert there is no significant difference between presentation conditions (*U* = 4350, *p* = 0.17).

These findings reflect that, at lest for these two performers: (1) the expert's motion was more consistent between trials *and* between the veridical and deceptive presentations, (2) the novice's motion was more variable overall and (3) there was significant motion variability between the veridical and performance conditions.

### 5.3. Grasp force

Finally, we investigate the grasping behavior of the two performers. Anecdotal evidence suggests that tension transfer is a crucial element of the FDS deception (Teller, Personal Communication) and empirical results Cavina-Pratesi et al. (2011) further support the notion that magicians' grasp can have an effect on the perception of sleight-of-hand performances.

During an effective performance of the FDS the muscular tension needed to hold the coin in one hand is apparently “transferred” to the grabbing hand. Here we consider the act of simulating (or exaggerating) the muscle tension and its effects on the performance success of the two magicians.

#### 5.3.1. Apparatus and material

A BIOPAC (BIOPAC, Inc.) amplifier / data acquisition system, connected to a Macintosh Mac Book Pro running Mac OS 10.8 was used to collect the EMG data.

Each magician was outfitted with three electrodes on the anterior side of each forearm. The placement of the electrodes was based on the location of the *flexor digitorum superficalis* muscle and surrounding flexor muscles (Hoozemans and van Dieën, 2005). This corresponded with two electrodes on the upper wrist, one on the distal medial wrist, and one proximal on the lateral side. A third electrode was secured proximally on the forearm as a baseline to eliminate noise during the EMG recording. Finally, the electrodes and their leads were wrapped with a neutral colored Ace bandage, along the upper forearm, to limit their movement and potential for distraction. The performers' hands remained unobstructed and unencumbered.

#### 5.3.2. Procedure

As with the motion tracking, each magician performed twenty deceptive and twenty veridical trials in a random interleaving. By randomly specifying the trials we hoped to avoid a patterned, stereotypical motion as a result of repetitively performing the same task.

### 5.4. Results and discussion

**Figure 6:**
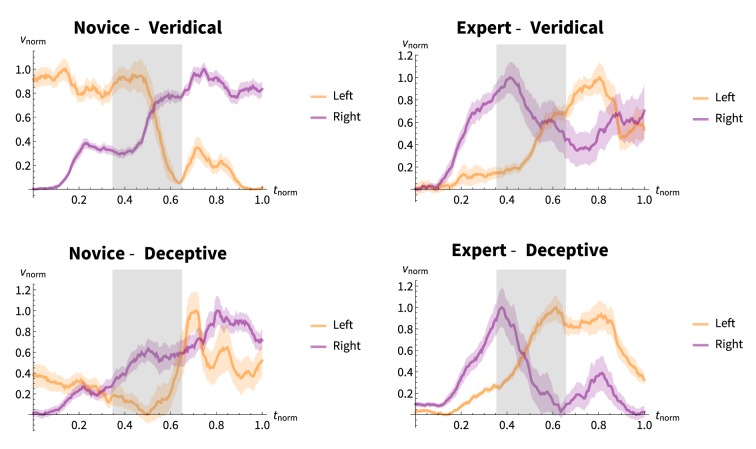
**Normalized EMG voltages for the magicians according to performance condition**. Orange and purple lines show mean normalized voltages for the electrode on the left- and right-arm *Flexor digitorum superficiallis*, respectively, across 20 trials. Bands surrounding the lines represent ± 1 SE. The capture phase is denoted by the gray box. The novice-veridical condition shows a stereotypical EMG response for the assumed FDS behavior. That is, the left hand initially grasps the coin and relaxes when the right hand grabs it.

EMG results are shown in Figure 6. As with the motion experiments, the individual trials were normalized on a time interval of *t_norm_* = [0, 1] and the EMG voltages for each *flexor superficiallis* resampled. Unlike the trajectory, we have also renormalized the EMG voltages. This is due to changing skin conductance and other difficult to control variation sources. These result in a wide variation of the the absolute voltages commensurate with grasping and releasing. For this, we used the “baselines” of a relaxed grasping finger pose, with and without the coin present. These are reflected by a *v_norm_* = 0.0 for the relaxed grasp and a *v_norm_* = 1.0 for maximum grasp.

The novice-veridical condition shows a stereotypical EMG response for the assumed FDS behavior. That is, the left hand initially grasps the coin and relaxes when the right hand grabs it. The right hand is initially relaxed and increases with tension after grasping. For the novice, there is a change in the behavior of the right hand in the deceptive condition from its behavior in the veridical condition. A post-experiment debriefing of the expert magician revealed that the idea of tension transfer was presented as part of the novice's training. It appears that the novice is trying but failing to execute this aspect of the FDS.

The expert has a non-stereotypical response in both the veridical and deceptive conditions. The trials start off relatively relaxed, then there is a small amount of a pre-flexing of the right hand with a subsequent relaxation and increasing of tension in the left hand. Note that, at the finish the right hand is more tense in the veridical condition, presumably because it is holding the coin, whereas this is not the case in the deceptive condition. This response suggests an exaggeration of the muscle tension since, at *t_norm_* = 0.0 the grasp force is, by definition, sufficient to hold the coin. As the trial proceeds, the coin is grasped more firmly before the capture-phase, and the subsequent retreat-phase shows this exaggeration as well.

**Figure 7:**
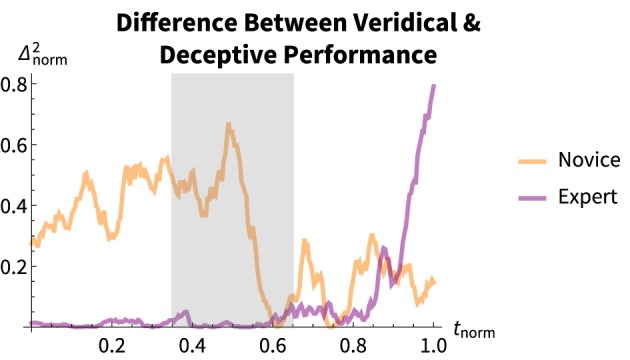
**Mean squared difference of the normalized grip voltage between the deceptive and veridical conditions for each magician**. The novice magician has a higher overall difference throughout the trick, with the exception at the capture-phase whereas the expert has little difference between the two grasp magnitudes until the very end of the performance.

It is most informative to examine the *difference* between the deceptive and veridical conditions. Presumably, in order to hide the result the magician should have as little difference as possible between the performance conditions. We took the squared difference of the normalized EMG voltage at each timepoint in the performance, shown in Figure 7.

The novice magician has a significantly higher overall difference throughout the trick (with the exception of a brief instant during the capture-phase) whereas the expert has little difference between the two grasp magnitudes until the very end of the performance. This is reflected in the overall difference *Mdn_novice_* = 0.31 vs. *Mdn_expert_* = 0.02, *U* = 8220, *p* < 0.0001, *r* = 0.90.

Only the novice's veridical condition shows a stereotypical grasping result. The novice's deceptive motion and both of the expert's performance conditions show some other behavior—but, the expert is consistent across both conditions with the exception of the very end of the retreat-phase.

## 6. Discussion

Experiments 1 and 2 show a fundamental effect for skill level of the French Drop Sleight and isolate the point in the motion where the deception takes place. Variability of observers' detection is greatest during the capture phase of the motion, and to a lesser degree, in the retreat phase. This indicates that aspects of the intention of the motion are likely revealed during the capture phase and to a lesser extent, the retreat phase. On a phenomenological level, one would intuitively assume the deception to occur during the mid-capture phase given that is where the mechanics of the illusion takes place. Conversely, the approach- and retreat-phases are relatively passive and therefore should reveal little about the location of the coin. In fact, as Experiment 2 showed, there *is* something informative occurring during the retreat phase related to the deception. Our results show that the novice is signaling his intention, in some form, during the retreat phase in addition to the mid-capture phase.

To examine the nature of the biological motion of the performers, we further investigated the trajectory and grasp for each magician in our experiment. Ideally, one would assume minimal differences between veridical trials and deceptive trials. Consistent differences could possibly indicate a deception or “tell.” As expected, the novice magician's trajectory was more variable than the expert, and significantly different between veridical and deceptive trials. The expert magician performed the FDS with a more compact, economical motion that did not significantly vary between veridical and deceptive trials.

The grip tension in the hand is derived from contraction and relaxation of the *flexor digitorum superficialis* muscle, located in the forearms. A more convincing illusion is thought to rely on a realistic appearing transfer of grip tension between the hands. While our novice failed to smoothly achieve this, the expert showed an similar transfer of grip tension between the hands in both the veridical and deceptive case. Interestingly, the transfer wasn't what one would stereotypically expect when moving an object from one hand to the other, but rather was exaggerated, perhaps as an effort to “sell” the deception.

It is crucial to note two things about our kinematic and muscular findings. First, this is obviously not a representative sample of magicians or FDS performance techniques. The fact that the expert taught the novice ensured some degree of consistency in attempted performance, yet there is certainly more variability to be had in the performance of the FDS. Therefore, it is crucial to not generalize these findings. Second, it is not clear that these kinematic or muscular variations are perceptible by human observers. We present them not as a final explanation of the sources of the detectability but as a suggestion for areas that need further study. One such approach for the kinematic data might take the form used in Diaz et al. (2012) where a minimal representation of the motion is presented (point-light display) with components of the motion systematically masked. The relative detectability of the deception in each case reveals facets of the motion crucial for the deception.

Taken together, the results from these experiments help to uncover the elements which contribute to the successful biological illusionary motion contained in the FDS. Clearly social cues and misdirection play a role in deceptive biological motion as a whole, but such overt clues do not fully explain the psychophysical manifestation of the deception.

## 7. Conclusion

The current study aimed to identify, isolate, extract, and measure the elements which contribute to the deception demonstrated in the French Drop Sleight of hand illusion. We demonstrated an effect for skill level of magician, highlighted where in the motion the deception occurs, and suggest biomechanical mechanisms contributing to the deception. For these two magicians, the combination of exaggerated tension transfer and a smooth and consistent trajectory path play a significant role in the FDS illusion.

### 7.1. Human subjects

This research was approved by the Skidmore College Participant Review Board.

### 7.2. Data sharing

The raw data, *Mathematica* and R analyses are available from the corresponding author and on-line at https://academics.skidmore.edu/blogs/flip/.

## Author contributions

Skidmore Student Opportunity Funds (MN and EE).

## Conflict of interest statement

The authors declare that the research was conducted in the absence of any commercial or financial relationships that could be construed as a potential conflict of interest.

## References

[B1] Abernethy B. (2008). Anticipation in squash: differences in advance cue utilization between expert and novice players. J. Sports Sci. 8, 17–34. 10.1080/02640419008732128 2359149

[B2] Arendsen J. van Doorn A. J. de Ridder H. (2007). When and how well do people see the onset of gestures? Gesture 7, 305–342. 10.1075/gest.7.3.03are9507702

[B3] Barnhart A. S. (2010). The exploitation of gestalt principles by magicians. Perception 39, 1286–1289. 10.1068/p6766 21125955

[B4] Beth T. Ekroll V. (2014). The curious influence of timing on the magical experience evoked by conjuring tricks involving false transfer: decay of amodal object permanence? Psychol. Res. [Epub ahead of print]. 10.1007/s00426-014-0584-2 24941913

[B5] Binet A. (1894). The psychology of prestidigitation, in Annual Report of the Board of Regents of the Smithsonian Institution (Washington, DC: US Government Printing Office), 555–571.

[B6] Cavina-Pratesi C. Kuhn G. Ietswaart M. Milner A. D. (2011). The magic grasp: motor expertise in deception. PLoS ONE 6:e16568. 10.1371/journal.pone.0016568 21347416 PMC3036651

[B7] Cui J. Otero-Millan J. Macknik S. L. King M. Martinez-Conde S. (2011). Social misdirection fails to enhance a magic illusion. Front. Hum. Neurosci. 5:103. 10.3389/fnhum.2011.00103 22046155 PMC3202226

[B8] Diaz G. J. Fajen B. R. Phillips F. (2012). Anticipation from biological motion: the goalkeeper problem. J. Exp. Psychol. Hum. Percept. Perform. 38, 848–864. 10.1037/a0026962 22309088

[B9] Farrow D. Abernethy B. (2003). Do expertise and the degree of perception - action coupling affect natural anticipatory performance? Perception 32, 1127–1139. 10.1068/p3323 14651325

[B10] Hoozemans M. J. M. van Dieën J. H. (2005). Prediction of handgrip forces using surface EMG of forearm muscles. J. Electromyogr. Kinesiol. 15, 358–366. 10.1016/j.jelekin.2004.09.001 15811606

[B11] Huys R. Smeeton N. J. Hodges N. J. Beek P. J. Wiliams A. M. (2008). On the dynamic information underlying visual anticipation skill. Percept. Psychophys. 70, 1217–1234. 10.3758/PP.70.7.1217 18927005

[B12] Hyman R. (1989). The psychology of deception. Annu. Rev. Psychol. 40, 133–154 10.1146/annurev.ps.40.020189.001025

[B13] Jackson R. C. Warren W. H. Abernethy B. (2006). Anticipation skill and susceptibility to deceptive movement. Acta Psychol. 123, 355–371. 10.1016/j.actpsy.2006.02.002 16546104

[B14] Jastrow J. (1896). Psychological notes upon sleight-of-hand experts. Science 3, 685–689. 10.1126/science.3.71.685 17743833

[B15] Johansson G. (1973). Visual perception of biological motion and a model for its analysis. Percept. Psychophys. 14, 201–211. 10.3758/BF0321237815820512

[B16] Király I. Jovanovic B. Prinz W. Aschersleben G. Gergely G. (2003). The early origins of goal attribution in infancy. Conscious. Cogn. 12, 752–769. 10.1016/S1053-8100(03)00084-9 14656515

[B17] Kuhn G. Amlani A. A. Rensink R. A. (2008). Towards a science of magic. Trends Cogn. Sci. 12, 349–354. 10.1016/j.tics.2008.05.008 18693130

[B18] Kuhn G. Land M. F. (2006). There's more to magic than meets the eye. Curr. Biol. 16, R950–R951. 10.1016/j.cub.2006.10.012 17113372

[B19] Lamont P. Henderson J. M. (2009). More attention and greater awareness in the scientific study of magic. Nat. Rev. Neurosci. 10:241. 10.1038/nrn2473-c1 19172168

[B20] Lamont P. Wiseman R. (2005). Magic in Theory: An Introduction to the Theoretical and Psychological Elements of Conjuring. Hatfield, Hertfordshire: University of Hertfordshire Press.

[B21] Macknik S. L. King M. Randi J. Robbins A. Thompson J. Martinez-Conde S. (2008). Attention and awareness in stage magic: turning tricks into research. Nat. Rev. Neurosci. 9, 871–879. 10.1038/nrn2473 18949833

[B22] Michotte A. (1963). The Perception of Causality. New York, NY: Basic Books.

[B23] Müller S. Abernethy B. Farrow D. (2006). How do world-class cricket batsmen anticipate a bowler's intention? Q. J. Exp. Psychol. 59, 2162–2186. 10.1080/02643290600576595 17095494

[B24] Nelms H. (1969/2000). Magic and Showmanship: A Handbook for Conjurers. New York, NY: Dover.

[B25] Otero-Millan J. Macknik S. L. Robbins A. Martinez-Conde S. (2011). Stronger misdirection in curved than in straight motion. Front. Hum. Neurosci. 5:133. 10.3389/fnhum.2011.00133 22125518 PMC3221472

[B26] Possidente P. Phillips F. Matthis J. Diaz G. J. (2011). Anticipation of sabre fencing attacks. J. Vis. 11, 957–957 10.1167/11.11.957

[B27] Quinn P. C. Bhatt R. S. (2005). Good continuation affects discrimination of visual pattern information in young infants. Percept. Psychophys. 67, 1171–1176. 10.3758/BF03193550 16502839

[B28] Soechting J. F. Engel K. C. Flanders M. (2001). The Duncker illusion and eye-hand coordination. J. Neurophysiol. 85, 843–854. 11160517 10.1152/jn.2001.85.2.843

[B29] Thomas F. Johnston O. (1981). Disney animation: the illusion of life. 1st Edn. New York, NY: Abbeville Press.

[B30] Todorov E. Jordan M. I. (2002). Optimal feedback control as a theory of motor coordination. Nat. Neurosci. 5, 1226–1235. 10.1038/nn963 12404008

[B31] Troje N. F. (2002). Decomposing biological motion: a framework for analysis and synthesis of human gait patterns. J. Vis. 2, 371–387. 10.1167/2.5.2 12678652

[B32] Troje N. F. Westhoff C. Lavrov M. (2005). Person identification from biological motion: effects of structural and kinematic cues. Percept. Psychophys. 67, 667–675. 10.3758/BF03193523 16134460

[B33] Wexler M. Klam F. (2001). Movement prediction and movement production. J. Exp. Psychol. Hum. Percept. Perform. 27, 48–64. 10.1037/0096-1523.27.1.48 11248940

